# Anteromedial versus transtibial technique in single-bundle autologous hamstring ACL reconstruction: a meta-analysis of prospective randomized controlled trials

**DOI:** 10.1186/s13018-017-0671-3

**Published:** 2017-11-07

**Authors:** Haitao Chen, Kai Tie, Yongjian Qi, Bin Li, Biao Chen, Liaobin Chen

**Affiliations:** Department of Orthopaedic Surgery, Zhongnan Hospital of Wuhan University, Wuhan, 430071 China

**Keywords:** Anterior cruciate ligament, Reconstruction, Anteromedial, Transtibial, Meta-analysis

## Abstract

**Background:**

The aim of this study was to compare the clinical outcome and postoperative complication between single-bundle anterior cruciate ligament (ACL) reconstruction with an anteromedial (AM) technique and a transtibial (TT) technique.

**Methods:**

The study includes clinical randomized controlled trials comparing the clinical outcomes of ACL reconstruction using the autologous hamstring tendon with an AM method and a TT method published up to September 2017 were retrieved from PubMed, Cochrane Library, and Embase databases. Relevant data were extracted and the Physiotherapy Evidence Database (PEDro) scale was used to assess the methodological quality. Stata/SE 12.0 was used to perform a meta-analysis of the clinical outcome.

**Results:**

Five RCTs were included, with a total of 479 patients: 239 patients and 240 patients in the AM group and the TT group, respectively. Assessing postoperative stability, better results were found in the AM group for the negative rate of the Lachman test (*P* < 0.05), the negative rate of the pivot-shift test (*P* < 0.05) and the side-to-side difference (*P* < 0.05). Assessing postoperative functional outcome, the AM group yielded superior results in proportion with International Knee Documentation Committee (IKDC) grade A (*P* < 0.05) and the Lysholm scores (*P* < 0.05) but had a comparable IKDC score (*P* > 0.05). In terms of postoperative complication, no significant difference was found between the AM group and the TT group (*P* > 0.05).

**Conclusions:**

The outcome of single-bundle ACL reconstruction with the AM technique is better than that with the TT technique in terms of postoperative stability and functional recovery of the knee.

## Background

Anterior cruciate ligament (ACL) injury is known to be one of the most common sports injuries, and ACL reconstruction is widely used because of the low success rate of conservative treatment [1]. The major goals of ACL reconstruction are to reconstruct knee stability, recover the patient’s pre-injury sports capability, and control the long-term joint degeneration [2–5]. The single-bundle ACL reconstruction has long been the gold standard of ACL treatment [3, 6, 7]. In this meta-analysis, only the studies about single-bundle ACL reconstruction are included.

The success of ACL reconstruction surgery depends mainly on similarities between the graft morphology, tension, position, and orientation compared to the native ACL. Traditionally, a transtibial (TT) technique of the femoral tunnel is the most common method used in single-bundle ACL reconstruction [8–10]. However, recent studies have shown that using the TT technique may lead to nonanatomic [11, 12], usually anteriorly positioned femoral tunnels [5, 10, 13–15]. To address problems related to the TT technique, more attention has been paid to the anatomic and biomechanical factors to ensure a successful outcome in ACL reconstruction techniques [16]. An anteromedial (AM) technique, also known as a transportal (TP) technique [17, 18], is the most common type of anatomical ACL reconstruction, which is now gradually accepted and adopted by more surgeons to reconstruct ACL rupture [9, 19]. The TT technique and AM technique are now commonly used treatment strategies in restoring the stability and kinematics of the joint [20–22]; However, whether the AM technique can achieve better clinical outcome than the TT technique is controversial. Several studies have shown that the AM elicited greater knee stability and improved the functional outcomes [17, 18, 23, 24]. On the contrary, other researchers have claimed that no definitive evidence could conclude that the AM technique was superior to the TT technique [10, 25–29] and the former might increase several other complications [17, 30–33].

A recent systematic review and meta-analysis [16] concluded that the AM technique showed superior surgeon-recorded stability; however, no significant difference was found in patient-reported functional outcomes. As the studies included in the review were mostly retrospective cohorts, with low levels of evidence, it is necessary to update the literature and make a meta-analysis with a high evidence grade. In our present meta-analysis, only prospective randomized controlled trials (RCTs) were included to compare the clinical outcome between the AM and TT technique in single-bundle autologous hamstring ACL reconstruction.

## Methods

### Search strategy

PubMed, Cochrane Library, and Embase databases were searched from their earliest entries up to September 2017. A manual search of all reference lists contained in the literature was also performed. Search strategies were used with different combination of keywords: (“Randomized Controlled Trials” OR trial OR placebo OR groups OR controlled OR Random*) AND (TP OR transportal OR Transtibial OR “TT technique” OR AMP OR Anteromedial) AND (“Reconstructive Surgical Procedures” OR Arthroscopy OR “Joint instability” OR Reconstructions OR Laxity OR “ligament integrity” OR rotation OR “rotary motion” OR function) AND (“intra-articular knee ligament” OR “Anterior Cruciate Ligament” OR ACL).

### Inclusion criteria and exclusion criteria

Inclusion criteria were as follows: (1) subject- all adult patients who underwent arthroscopy-assisted ACL reconstruction, with no limitation to sex or race; (2) intervention method—comparison of clinical outcome between the AM and TT technique in single-bundle autologous hamstring ACL reconstruction; (3) outcome parameters—Lachman tests, pivot-shift tests, proportion with IKDC grade A, IKDC scores, Lysholm scores, side-to-side difference (SSD), and complications; (4) study type—prospective RCT.

The exclusion criteria were (1) non-prospective trials (e.g., retrospective studies, observational studies, case series, and reviews); (2) animal or cadaver studies; (3) comparisons that were not between AM and TT method in ACL reconstruction; (4) studies not with single-bundle ACL reconstruction; (5) studies using allograft, bone-patellar tendon-bone, or Achilles tendon; (6) studies with a low level of evidence; and (7) laboratory studies.

### Literature selection

All potential studies were imported into Endnote X7 and duplicates were excluded. Then, two researchers (HTC and KT) independently excluded studies based on titles and abstracts. At last, by reading the full text carefully, the two researchers eliminated the studies that did not satisfy the selection criteria. Disagreements were resolved by discussion with the corresponding researcher (LBC).

### Data extraction and assessment of study quality

Two researchers (HTC and KT) independently checked all potentially suitable studies using a pre-designed sheet to perform data extraction. Any disagreements were resolved by discussion. Extracted data included article information (author and publication date), participant demographics, follow-up period, sample size, implant, fixation type, outcome parameter, and postoperative complication. Some omitted data such as the mean and standard deviations of the Lysholm scores in Noh’s study [1] are estimated according to a specific method [34] because the original data is unavailable.

Working independently, the same two researchers assessed the study quality according to The Physiotherapy Evidence Database (PEDro) scale, which comprises 11 items based on the Delphi list, was used to assess the methodological quality of each article [35]. Each item was scored yes or no, with a maximum score of 10 because criterion one was not scored. A trial with a score of ≥ 6 was considered to be of high quality.

### Statistical analysis

The meta-analysis was conducted using Stata/SE version 12.0. All extracted data were checked and input by reviewers. When the outcome indicator was dichotomous outcomes, relative risk (RR) was calculated for effect size. For continuous outcomes, a weighted mean difference (WMD) was calculated when the same measurement criterion was used; otherwise, a standardized mean difference (SMD) was calculated. Both used 95% confidence intervals (CI). The intervening effect of an indicator was considered as zero difference if 95% CI for WMD or SMD contained 0 and 95% CI for RR contained one. The statistical heterogeneity was tested with the chi-square test and *I* ^2^. If heterogeneity was low (*P* > 0.1 or *I* ^2^ ≤ 50%), a fixed-effects model was used. If heterogeneity was significant (*P* < 0.1, *I* ^2^ > 50%), sensitivity analysis, subgroup analyses, and meta-regression were conducted to find the source of the heterogeneity. If the heterogeneity could not be eliminated, a random-effects model would be used when the result of meta-analysis had clinical homogeneity, or descriptive analysis would be used. Begg’s test was used to check the publication bias of involved articles.

## Results

### Search results

**Fig. 1:**
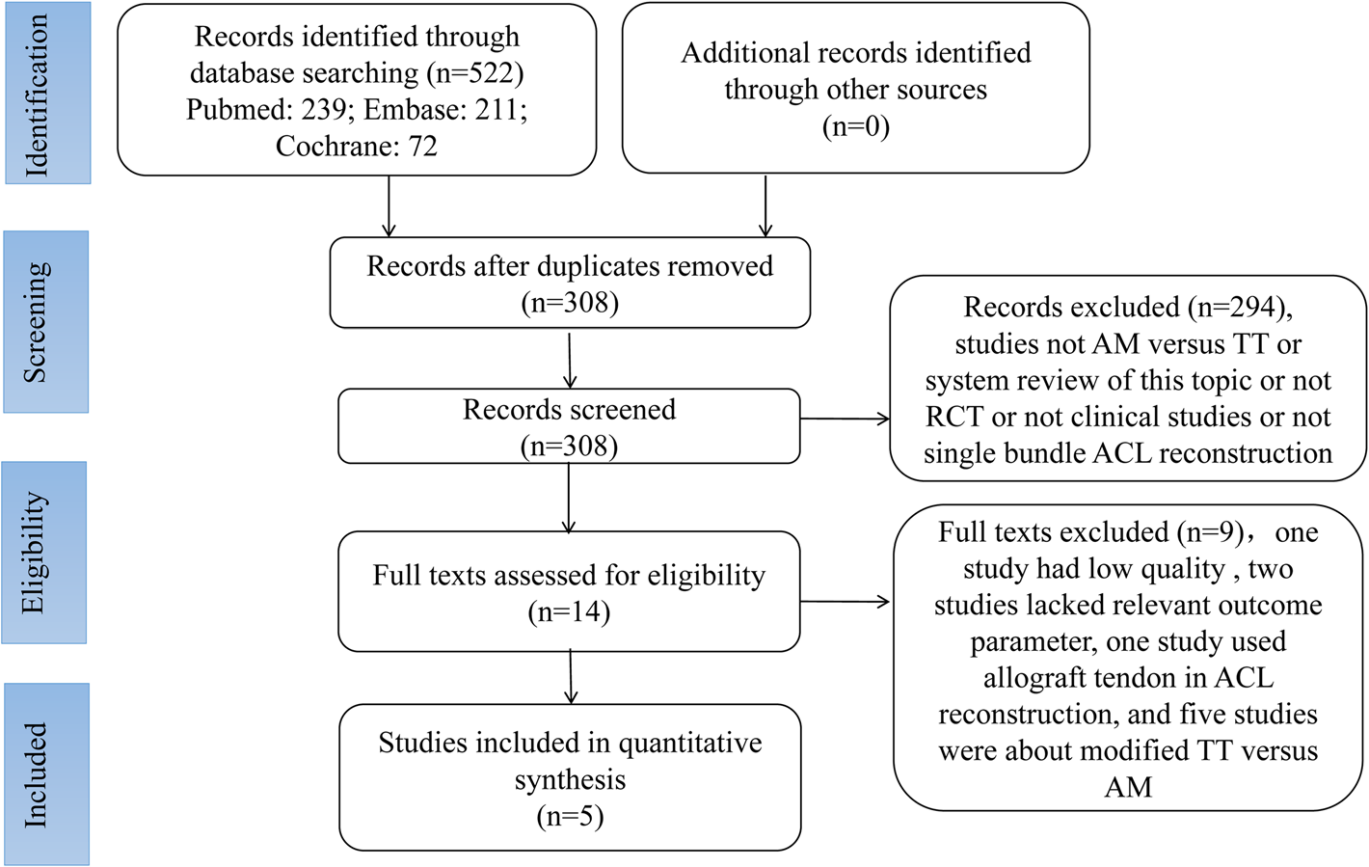
Flowchart of article selection process

Five-hundred twenty-two relevant articles were initially selected according to the search strategy. Two-hundred fourteen were excluded after checking for duplicates with the literature management software Endnote X7. Two-hundred ninety-four were excluded after reviewing the titles and the abstracts, nine published articles were excluded by reviewing their full content as one study had low quality, two studies lacked relevant outcome parameter, one study used allograft tendon in ACL reconstruction, and five studies were about modified TT versus AM. Finally, five articles [9, 13, 14, 36, 37] were included in the meta-analysis. A summary of the review process is presented in Fig. 1.

### Description of included studies

All five selected articles were written in English, which compared the clinical outcomes of the AM and TT techniques in ACL reconstruction. The implants were all autologous hamstrings, with different fixation methods, and all follow-up periods were ≥ 6 months. There was a total of 479 patients: 239 patients and 240 patients in the AM group and the TT group, respectively. All basic article information is reported in Table 1, and the postoperative outcome measures of the two techniques are reported in Table 2. All of the five selected articles were RCTs and assessed using the PEDro scale. The results showed that all articles scoring ≥ 6 were of high quality. The methodological score of each included RCT with general remarks is shown in Table 3.

**Table 1:** Description of included trials

Author	Year	Age (years)	Follow-up (months)	Number of patients		Implant	Fixation type	Outcome	Postoperative complication
				AM	TT				
Bohn.	2015 [36]	AM: 24.3 ± 4.9 TT: 27.5 ± 7.2	12–18	12	11	HT	EB + BS	Lachman test; PS test; KT1000 (SSD); IKDC grades; IKDC scores; KOOS4; Tegner scores; Lysholm scores; 3-D motion analysis	–
Guglielmetti	2014 [13]	< 40	6	38	35	HT	ETD + MIS	Anterior drawer test; Lachman test; PS test; SSD; IKDC grades; length of the femoral tunnel	AM: superficial infection, mobility deficits, and arthrofibrosis
Hussein	2012 [14]	AM: 34.2 TT: 32.6	AM: 50.5 TT: 52	78	72	HT	SF + BS	SSD; PS test; Lysholm scores; IKDC scores; IKDC grades	–
Mirzatolooei	2012 [9]	AM: 26.6 TT: 26.8	> 18	80	88	HT	TransFix	IKDC grades; Lysholm scores; Lachman test; PS test; SSD	AM: saphenous nerve injury TT: saphenous nerve injury, septic arthritis
Zhang	2012 [37]	28	> 12	31	34	HT	Rigidfix + Intrafix	Lysholm scores; KT-1000 (SSD)	–

*AM* anteromedial, *TT* transtibial, *HT* hamstring tendon, *EB* Endobutton, *BS* Bio-interference screw, *ETD* the Endo Tunnel Device, *MIS* metal interference screw, *SF* suspensory fixation, *PS* pivot-shift, *IKDC* International Knee Documentation Committee, *KOOS* Knee Injury and Osteoarthritis Outcome Score, *SSD* side-to-side difference

**Table 2:** Postoperative outcome measures of AM group Versus TT group

Study	N	Lachman Test (N/P)		PS Test (N/P)		IKDC A (Y/N)		IKDC scores		Lysholm scores		SSD (mm)		Postoperative Complication (Y/N)	
		AM	TT	AM	TT	AM	TT	AM	TT	AM	TT	AM	TT	AM	TT
Bohn (2015) [36]	23	9/3	8/3	10/2	8/3	3/9	3/8	76 ± 13	71 ± 15	86 ± 12	81 ± 14	2.0 ± 1.7	2.3 ± 1.9	–	–
Guglielmetti (2014) [13]	73	33/5	25/10	33/5	26/9	28/10	18/17	–	–	–	–	–	–	2/36	0/35
Hussein (2012) [14]	150	–	–	52/26	30/42	69/9	57/15	90.6 ± 6.4	90.2 ± 7.6	91.8 ± 4.3	90.9 ± 7.0	1.6 ± 0.8	2.0 ± 0.9	–	–
Mirzatolooei (2012) [9]	168	70/10	68/20	70/10	70/18	–	–	–	–	81.41 ± 8.2	78.32 ± 10.7	1.73 ± 0.85	2.2 ± 1.13	2/78	4/84
Zhang (2012) [37]	65	–	–	–	–	–	–	–	–	95.1 ± 1.0	94.5 ± 1.1	1.96 ± 1.02	2.14 ± 0.91	–	–

*AM* anteromedial, *TT* transtibial, *N/P* negative/positive, *Y/N* yes/no, *PS* pivot-shift, *IKDC* International Knee Documentation Committee, *SSD* side-to-side difference

**Table 3:** PEDro critical appraisal tool results

Study	Criteria											Total
	1	2	3	4	5	6	7	8	9	10	11	
Bohn et al	✓	✓	✓	✓	✓	✗	✓	✓	✓	✓	✓	9
Guglielmetti et al	✓	✓	✓	✓	✗	✗	✗	✓	✓	✓	✓	7
Hussein et al	✓	✓	✗	✓	✗	✗	✓	✓	✓	✓	✓	7
Mirzatolooei et al	✓	✓	✗	✓	✗	✗	✓	✓	✓	✓	✓	7
Zhang et al	✓	✓	✗	✓	✓	✗	✗	✓	✓	✓	✓	7

✓ Satisfied criterion, ✗ Did not satisfy criterion

Criteria: 1. Eligibility criteria were specified; 2. subjects were randomly allocated to groups (in a crossover study, subjects were randomly allocated an order in which treatments were received); 3. allocation was concealed; 4. the groups were similar at baseline with respect to the most important prognostic indicators; 5. all subjects were blinded to the procedure; 6. all therapists who administered the therapy were blinded; 7. all assessors who measured at least one key outcome were blinded; 8. measures of at least one key outcome were obtained from ≥85% of the subjects initially allocated to groups; 9. all subjects for whom outcome measures were available received the treatment or control condition as allocated or, where this was not the case, data for at least one key outcome was analyzed by intention to treat; 10. the results of between-group statistical comparisons are reported or at least one key outcome; 11. the study provides both point measures and measures of variability for at least one key outcome

### Lachman test

**Fig. 2:**
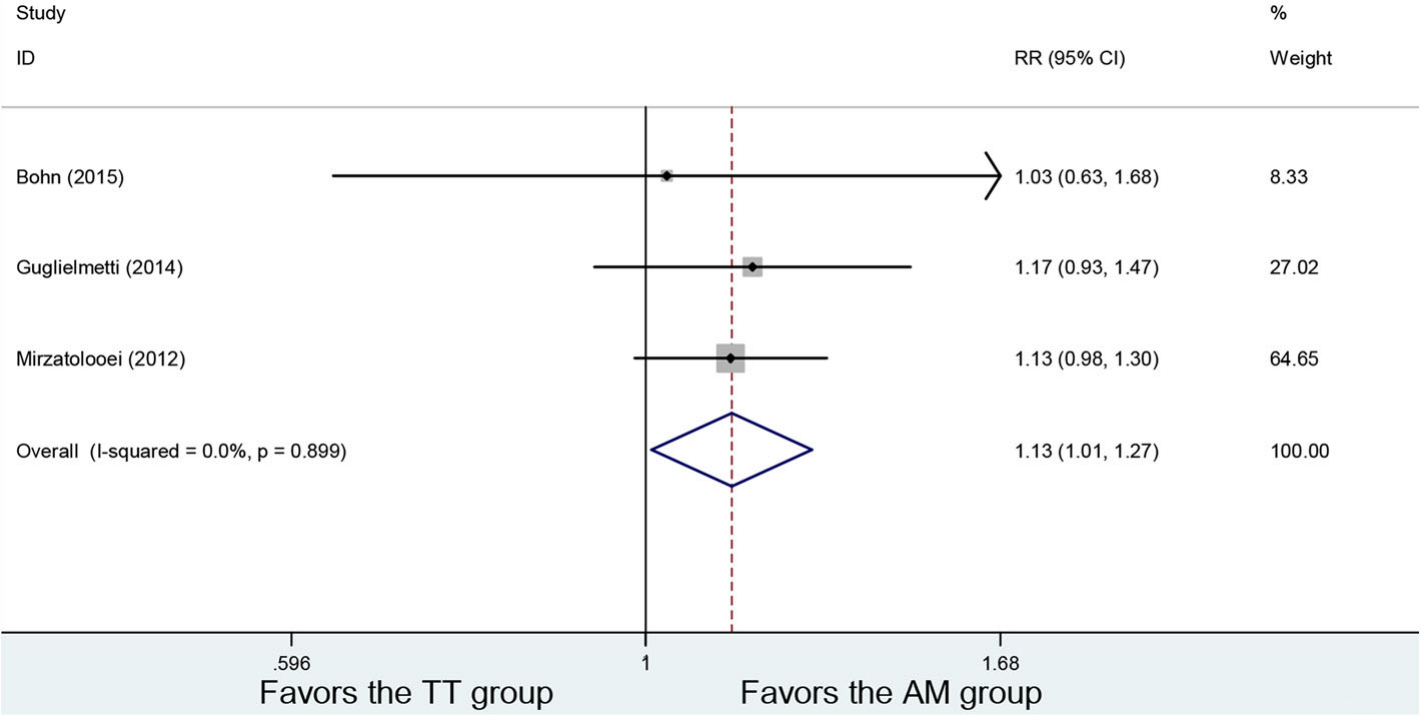
Forest plot of negative Lachman test

Postoperative Lachman tests were conducted in three studies. No heterogeneity was found among the studies (*P* = 0.899, *I* ^2^ = 0%). The postoperative negative Lachman test of 130 patients in the AM group and 134 patients in the TT group was analyzed using a fixed-effects model. The result showed a difference in Lachman test between the two groups (RR = 1.13, 95% CI (1.01, 1.27), *P* = 0.036). The AM group had a higher negative rate in Lachman test (Fig. 2).

### Pivot-shift test

**Fig. 3:**
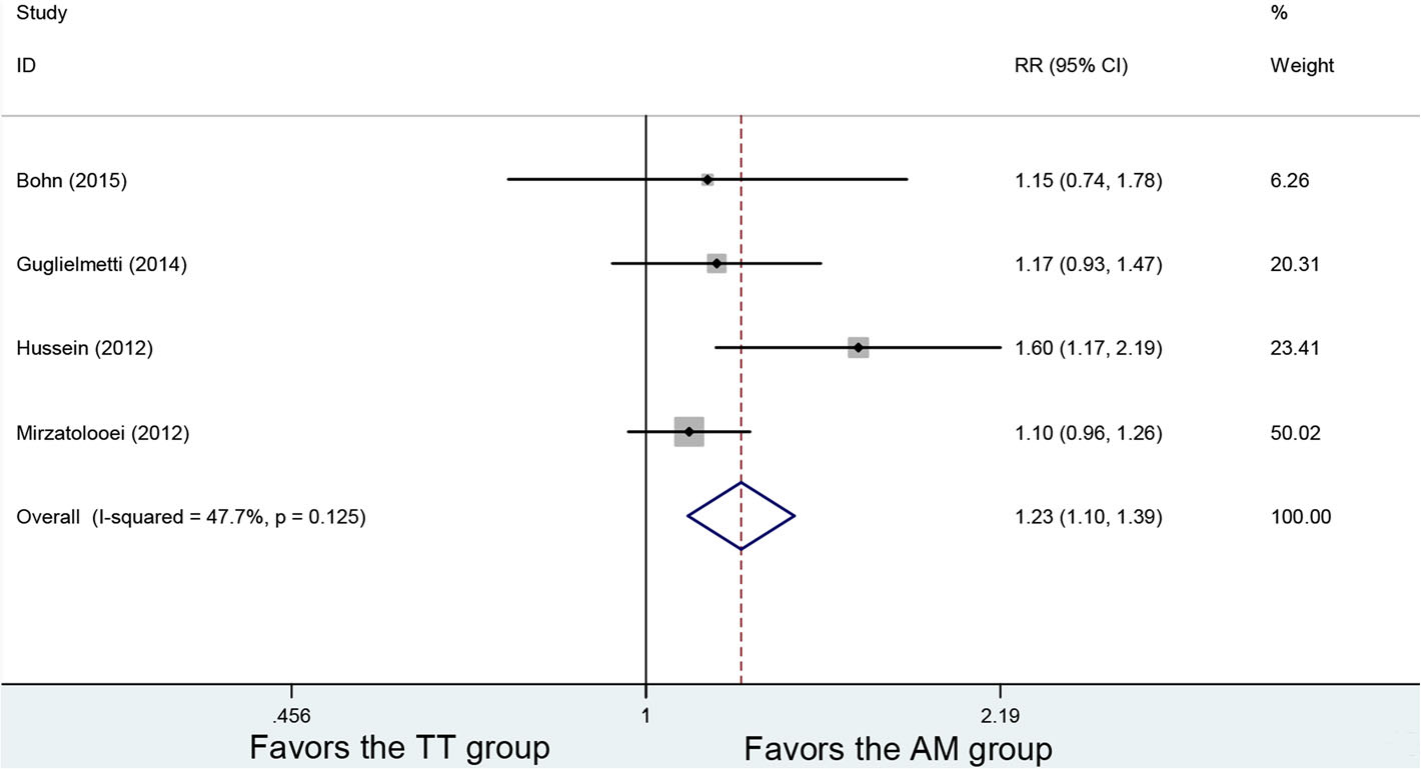
Forest plot of negative pivot-shift test

Postoperative Pivot-shift tests were conducted in four studies. The analysis of negative pivot shift results showed no heterogeneity among the studies (*P* = 0.125, *I* ^2^ = 47.7%). The postoperative negative pivot-shift of 208 patients in the AM group and 206 patients in the TT group were analyzed using a fixed-effects model, with a significant difference between the two methods (RR =1.23, 95% CI (1.10, 1.39), *P* = 0). The AM group had a higher negative rate in Pivot-shift test (Fig. 3).

### IKDC grades

**Fig. 4:**
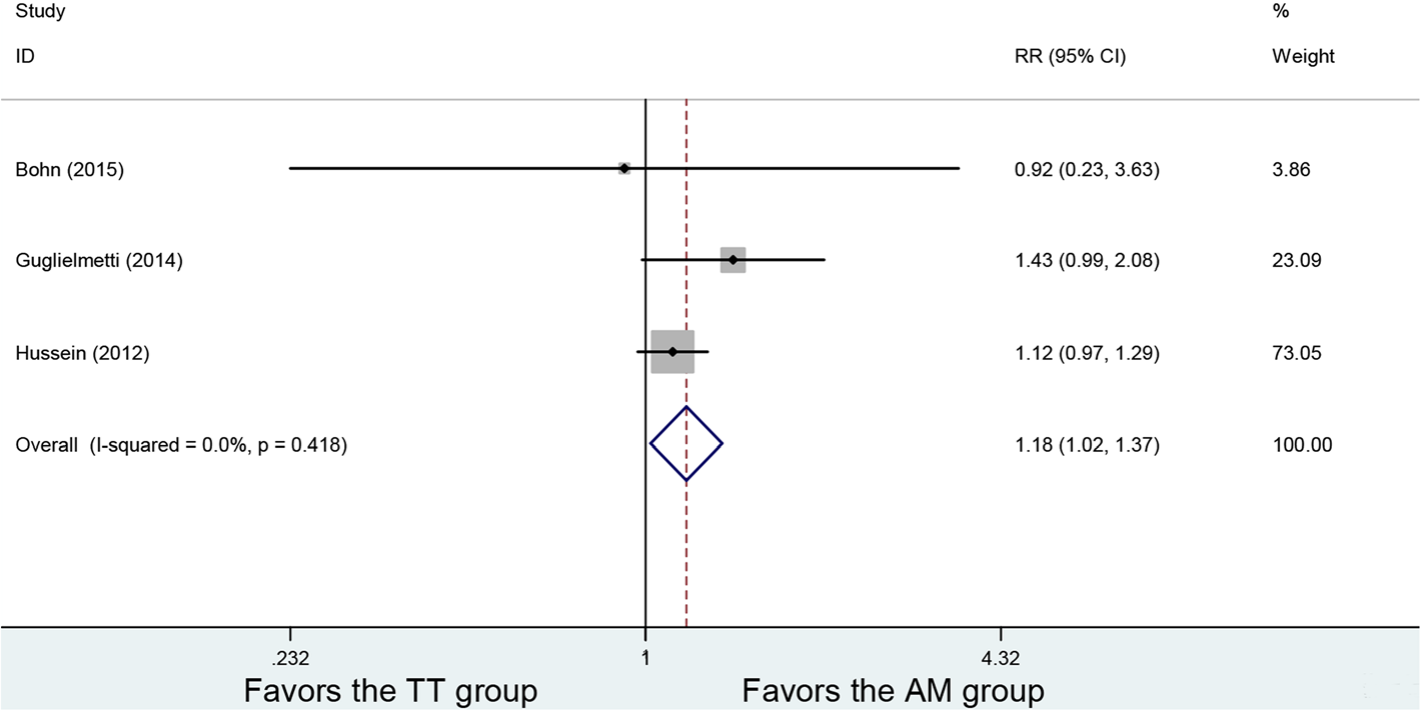
Forest plot of IKDC grades. WMD, weighted mean difference

Three studies included IKDC grades, and no heterogeneity was found among the studies (*P* = 0.418, *I* ^2^ = 0%). The 128 patients in the AM group and 118 patients in the TT group were analyzed using the fixed-effects model. Significant difference can be found between the two groups (RR = 1.18, 95% CI (1.02, 1.37), *P* = 0.025). The AM group had a higher proportion with IKDC grade A (Fig. 4).

### IKDC scores

**Fig. 5:**
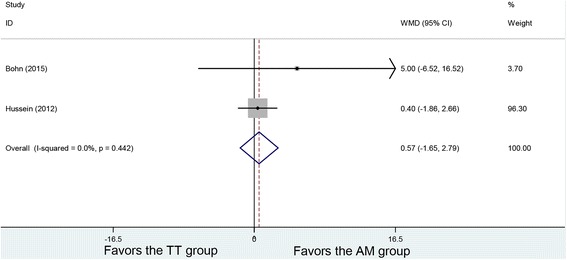
Forest plot of IKDC scores

Two studies demonstrated postoperative IKDC scores, with no heterogeneity being found among the studies (*P* = 0.442, *I* ^2^ = 0%). Using the fixed-effects model, 90 patients in the AM group and 83 patients in the TT group were analyzed with no significant difference in the postoperative IKDC scores (WMD = 0.57, 95% CI (− 1.65, 2.79), *P* = 0.614) (Fig. 5).

### Lysholm scores

**Fig. 6:**
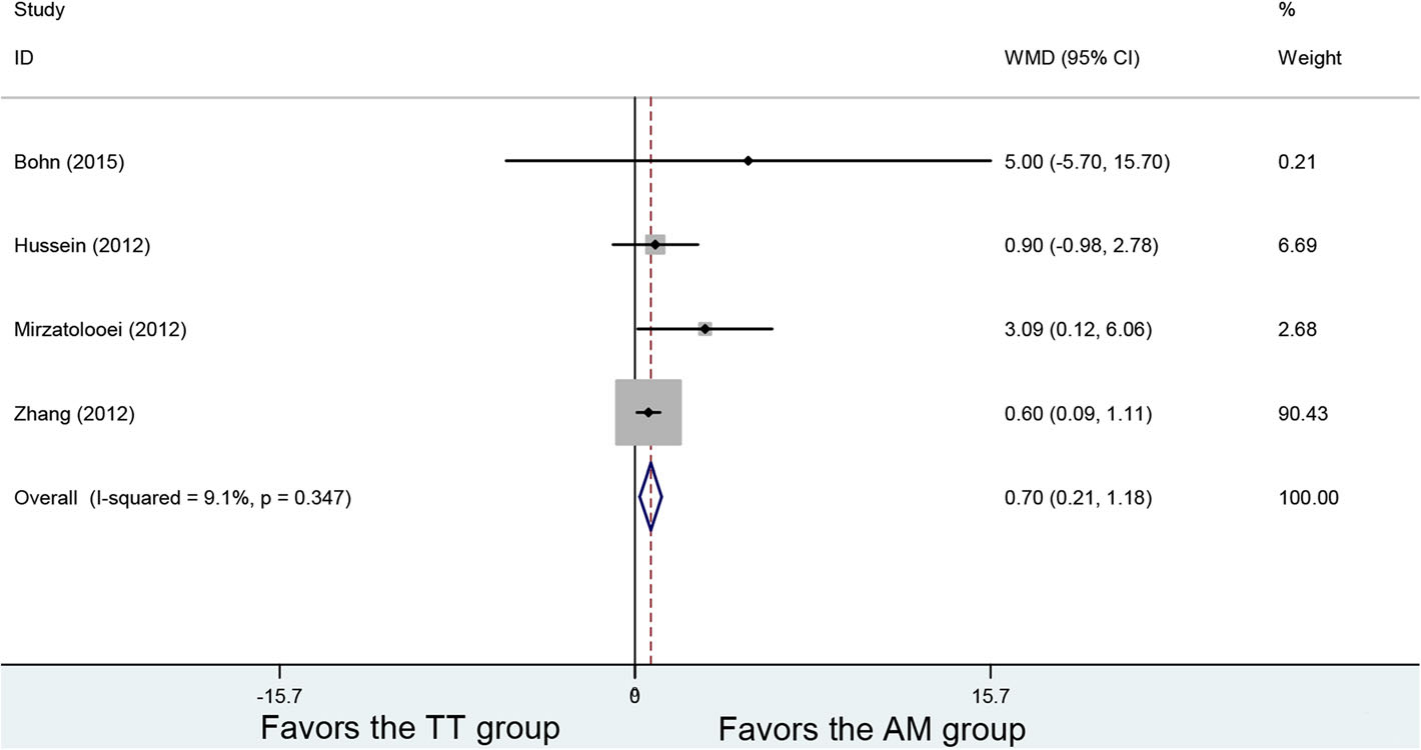
Forest plot of Lysholm scores

Four studies reported postoperative Lysholm scores. No heterogeneity was found among the studies (*P* = 0.347, *I* ^2^ = 9.1%). Using the fixed-effects model in the analysis, with 199 patients in the AM and 197 patients in the TT group, the result showed a difference in Lysholm scores between the two groups (WMD = 0.70, 95% CI (0.21, 1.18), *P* = 0.005). The AM group had higher Lysholm scores (Fig. 6).

### SSD

**Fig. 7:**
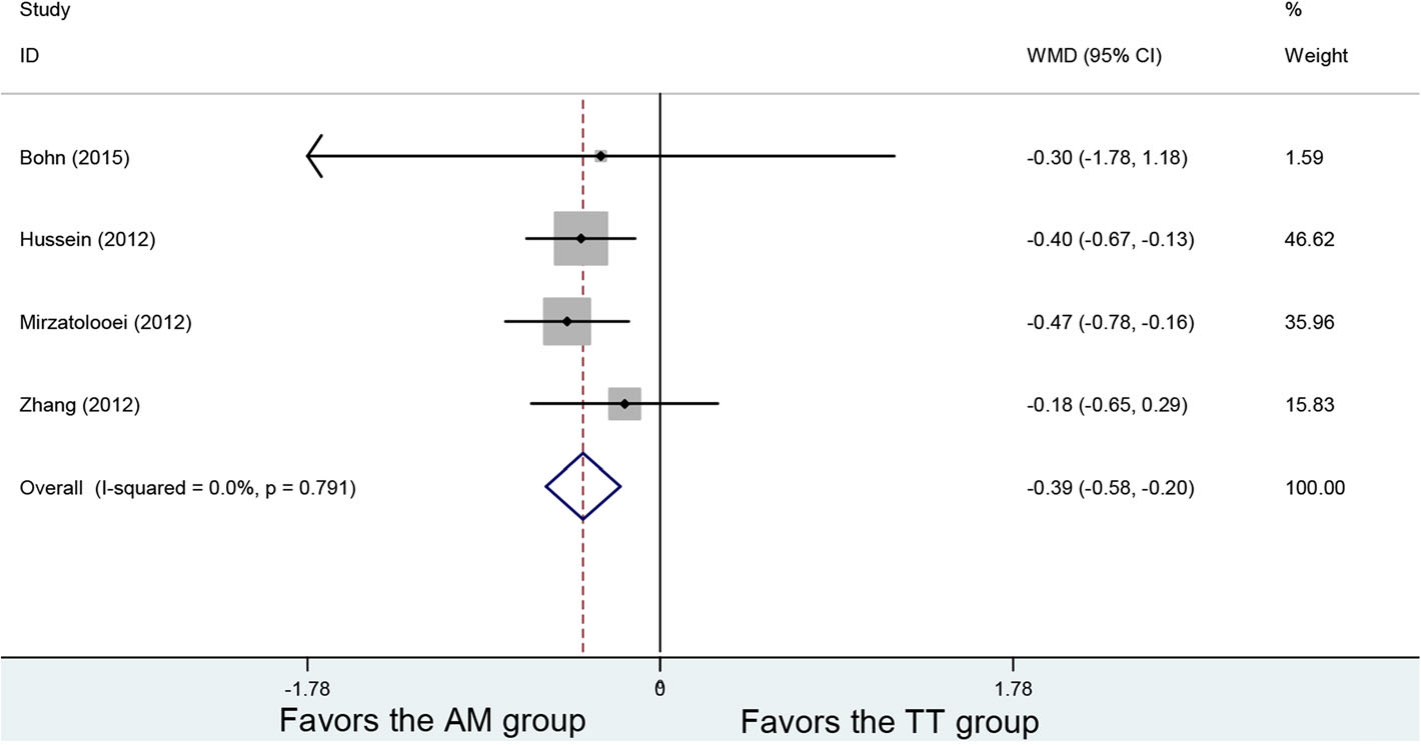
Forest plot of SSD

Four studies reported postoperative SSD. No heterogeneity was found among the studies (*P* = 0.791, *I* ^2^ = 0%). Using the fixed-effects model in the analysis, with 202 patients in the AM and 194 patients in the TT group, the result showed a difference in SSD between the two groups (WMD = − 0.39, 95% CI (− 0.58, − 0.20), *P* = 0). The TT group had higher SSD (Fig. 7).

### Postoperative complication

**Fig. 8:**
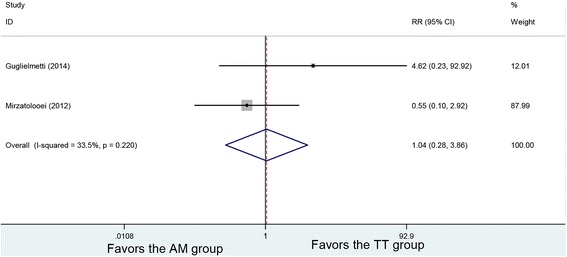
Forest plot of postoperative complication

Two studies reported postoperative complication. No heterogeneity was found among the studies (*P* = 0.22, *I* ^2^ = 33.5%). Using the fixed-effects model in an analysis, 118 patients in the AM and 124 patients in the TT group were analyzed with no significant difference in the postoperative complication (RR = 1.04, 95% CI (0.28, 3.86), *P* = 0.955) (Fig. 8).

### Publication bias

**Fig. 9:**
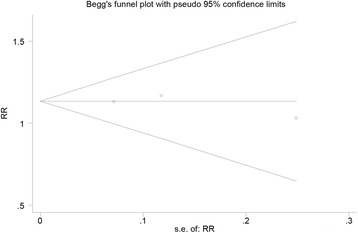
Funnel plot of publication bias for negative Lachman test

For Lachman test, used as an indicator in most studies as an example, Begg’s test was used to access the publication bias, showing the lack of bias among the included studies (Begg’s test, *P* = 1, Fig. 9).

## Discussion

In our meta-analysis, the AM technique and the TT technique in single-bundle autologous hamstring ACL reconstruction were compared in terms of the clinical outcome and complication, and the result showed that the outcome of the ACLs reconstructed with the AM techniques was superior in terms of the stability and functional recovery of the knee.

In our study, the AM technique yielded superior results in the outcome of stability, such as SSD, the negative rate of Lachman, and the pivot-shift test. This indicates that the AM technique may enhance the biomechanical properties of the reconstructed ACLs. For postoperative functional status, the AM technique yielded superior results in proportion with IKDC grade A and Lysholm scores but had comparable IKDC scores. IKDC scores were found significantly better in the AM group compared with the TT group in Guglielmetti’s research [13], but the relevant data of the IKDC scores were incomplete and couldn't be taken in to account. IKDC scores were also found higher in the AM group in Hussein’s study [14] and Bohn’s study [36]; however, no significant difference was found between the two techniques. Overall, it can be found that the AM technique could achieve greater functional recovery in single-bundle ACL reconstruction. At this stage, it is clear that the AM technique is better in single-bundle ACL reconstruction in terms of stability and functional recovery of the knee.

Some reasons may account for this result. First of all, compared with the TT technique, the AM technique might be superior in positioning the ACL femoral tunnel at the center of the native ACL footprint [27, 38, 39] and probably allowed for the creation of the femoral tunnel independently in a more anatomic position [8]. Silva et al. declared that, compared with the TT technique, the AM technique places the femoral and tibial tunnels more centrally in the ACL footprint which may allow better control of the anteroposterior and rotational stability of the knee, therefore improving the clinical outcome in the long run [5]. Second, the AM technique can restore the ACL in the appropriate orientation similar to the native ACL, which can ensure a better postoperative knee function and restoration of the physiological kinematics. Riboh et al. thought that femoral tunnels in the AM group were more oblique in the sagittal and coronal planes, resulting in decreased resting graft tension, a closer approximation of natural graft forces during motion [22]. Alentorn et al. reported that the oblique 10 o’clock position was found to restore rotational knee stability better than the 11 o’clock position [15]. Mirzatolooei et al. concluded that the use of the AM method in a more oblique femoral tunnel demonstrated better short-term clinical results than the TT technique in ACL reconstruction [9].

Postoperative complications like superficial infection, arthrofibrosis, and septic arthritis were reported in two of the included studies [9, 13]. According to the result of the present study, an occurrence rate of postoperative complications was low in both of groups, and no great difference was found between the two groups. Another complication of the AM method in ACL reconstruction is a short femoral tunnel, which may be associated with lower graft healing rate as the graft has less handle on the short tunnel [1]. However, in the present research, only one of the included studies [13] compared the length of the femoral tunnel and the relevant data were incomplete, thus a meta-analysis of the length was unachievable.

A recent meta-analysis from Chen’s [16] showed that the AM technique may have superior stability, while no significant difference was found in functional outcome. Riboh’s meta-analysis [22] showed that no significant clinical differences were found between the two techniques. In our present research, patients in the AM group had a better result in both stability and functional recovery of the knee. Compared with the two studies, there are several highlights in our study. First, only prospective, randomized, controlled trials (RCTs) were included. Second, only studies with single-bundle ACL reconstruction were included, since ACL reconstruction with single-bundle or double-bundle may get different results [11, 20, 40–42]. Finally, only autologous hamstring tendons are used in the included studies, since allograft or other autologous tendons may also give rise to a heterogeneity of the results. In our opinion, the result of our study is more objective and accurate.

The limitations of this study were as follows. (1) The whole sample size was not large, and the outcome indicator was not unified, which may have influenced the outcome. (2) The follow-up duration in the studies was varied, which may not have been sufficiently homogeneous to evaluate the differences between the two techniques. (3) Outcome indicator like anterior drawer tests or the Tegner score was referred to, respectively, in only one of the included study, and could not be used as outcome parameter in the present study.

## Conclusion

The outcome of single-bundle ACL reconstruction with the AM technique is better than that with the TT technique in terms of stability and functional recovery of the knee.

## Abbreviations

ACL: Anterior cruciate ligament
AM: Anteromedial
CI: Confidence intervals
IKDC: International Knee Documentation Committee
PEDro: The Physiotherapy Evidence Database
RCT: Randomized controlled trial
RR: Relative risk
SMD: Standardized mean difference
SSD: Side-to-side difference
TP: Transportal
TT: Transtibial
WMD: Weighted mean difference
